# Concordance rate between oligoclonal bands and the Kappa index in patients with suspected multiple sclerosis (MS)

**DOI:** 10.1055/s-0044-1779690

**Published:** 2024-03-14

**Authors:** Renan Barros Domingues, Márcio Vega dos Santos, Daiane Salomão, Carlos Senne

**Affiliations:** 1 Senne Liquor Diagnóstico, São Paulo SP, Brazil.

**Keywords:** Multiple Sclerosis, Immunoglobulin kappa-Chains, Cerebrospinal Fluid, Oligoclonal Bands, Immunoglobulin Light Chains, Esclerose Múltipla, Cadeias kappa de Imunoglobulina, Líquido Cefalorraquidiano, Bandas Oligoclonais, Cadeias Leves de Imunoglobulina

## Abstract

**Background** Oligoclonal bands (OCBs) and Kappa free light chains (FLCs) in the cerebrospinal ﬂuid (CSF) are sensitive markers of intrathecal immunoglobulin (Ig)G synthesis in patients with multiple sclerosis.

**Objective** To evaluate the concordance rate between OCBCs and the Kappa index (KI) in patients with suspected multiple sclerosis (MS).

**Methods** Patients with suspected MS were referred to a specialized CSF laboratory as part of their diagnostic investigation. Paired CSF and serum samples were collected and submitted to detection of OCBs and determination of the KI. Positive and negative results were determined with both methods, and the percentage of agreement between them was established.

**Results** In total, 171 serum and CSF samples from 171 patients were included in the analysis. The mean age of the patients was of 40 ± 14.2 years; 18.9% of them were male, and 81.1% were female. The OCBs and KI presented concordant results in 161 (94.2%) samples: in 74 (43.3%), both were positive, and in 87 (50.9%), both were negative. In 10 cases, the results were discrepant: KI positive/OCB negative in 8 and OCB positive/KI negative in 2 cases.

**Conclusion** The KI and OCBs presented high concordance level. Currently, the detection of OCBs in the CSF is the standard method for MS diagnosis, but it is time-consuming, and its visual interpretation can be difficult. The results suggest that the KI is a good alternative for the detection of intrathecal immunoproduction in cases of suspected MS.

## Resumo

**Antecedentes** Bandas oligoclonais (BOCs) e cadeias leves de imunoglobulina ( *free light chains* , FLCs, em inglês) Kappa no líquido cefalorraquidiano (LCR) são marcadores sensíveis da síntese intratecal de imunoglobulina (Ig)G em pacientes com esclerose múltipla (EM).

**Objetivo** Avaliar a taxa de concordância entre BOCs e o índice Kappa (IK) em pacientes com suspeita de EM.

**Métodos** Pacientes com suspeita de EM foram encaminhados a um laboratório especializado em LCR como parte de sua investigação diagnóstica. Amostras pareadas de LCR e soro foram coletadas e investigadas quanto à presença de BOCs e submetidas à determinação do IK. Resultados positivos e negativos foram determinados com ambos os métodos, e estabeleceu-se o percentual de concordância entre eles.

**Resultados** Ao todo, 171 amostras de soro e LCR de 171 pacientes foram incluídas na análise. A média de idade dos pacientes foi de 40 ± 14,2 anos; 18,9% deles eram do sexo masculino, e 81,1%, do sexo feminino. Resultados concordantes entre as BOCs e o IK foram observados em 161 (94,2%) amostras: em 74 (43,3%), ambos foram positivos, e em 87 (50,9%), ambos foram negativos. Em 10 casos, os resultados foram discrepantes: IK positivo/BOC negativo em 8, e BOC positivo/IK negativo em 2.

**Conclusão** Observou-se alto nível de concordância entre o IK e as BOCs. A detecção de BOCs no LCR é atualmente o método padrão para o diagnóstico de EM, mas é demorado, e sua interpretação visual pode ser difícil. Os resultados sugerem que o IK pode ser uma alternativa para a detecção de imunoprodução intratecal em casos de suspeita de EM.

## INTRODUCTION

**Figure 1:**
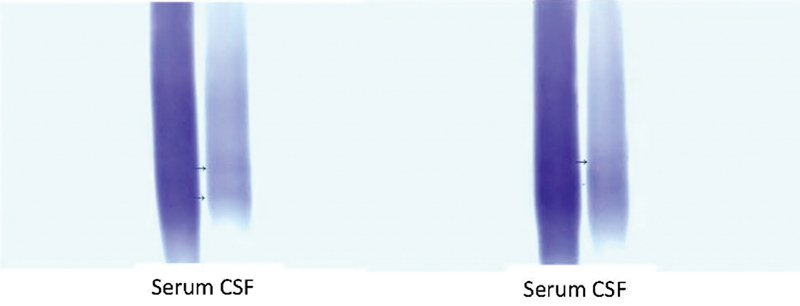
Two examples of detection of oligoclonal bands (OCBs) of difficult visual interpretation, obtained from paired samples of cerebrospinal fluid (CSF) and serum from patients with suspected multiple sclerosis (MS).

The assessment of intrathecal immunoglobulin G (IgG) production in the cerebrospinal fluid (CSF) is part of the diagnostic workup for multiple sclerosis (MS). 1 Currently, the standard test in this regard is the detection of oligoclonal bands (OCBs) in the cerebrospinal fluid (CSF) and serum via isoelectric focusing (IEF). 2 3 There are 5 different patterns in the detection of OCBs: type 1–no bands in the CSF and serum; type 2–OCBs only in the CSF; type 3–OCBs in the CSF and serum with additional bands in the CSF; type 4–identical OCBs in the CSF and serum; and type 5–monoclonal bands in the CSF and serum. Types 2 and 3 indicate intrathecal IgG synthesis. 3 The presence intrathecal synthesis by OCBs is currently included in the diagnostic criteria for MS. 4 However, IEF is a laborious and time-consuming technique that still presents the disadvantage of sometimes being difficult to be visually analyzed ( Figure 1 ), making it susceptible to misinterpretations.

While OCB detection is a qualitative method, there are other methods that quantitatively assess intrathecal IgG synthesis in the CSF, such as CSF IgG measure, IgG index, and Reiber nomogram. 5 6 More recently, the method of measuring CSF IgG free light chains (FLCs), especially the Kappa FLCs (KFLCs), has been assessed. 6 Several studies have reported that the nephelometric , turbidimetric, or enzyme-linked immunosorbent assay (ELISA) methods to determine FLCs are comparable to those used for OCB detection, in addition to being more time-efficient. 7 8 9 10 11 12 However, there are questions that are still unanswered: can the measurement of CSF IgG FLCs be an alternative to OCB detection? Can the diagnostic sensitivity be increased by performing these two methods in parallel? Moreover, there are still concerns related to the reproducibility of the Kappa index (KI), considering the different formulae used, with varying cut-off values adopted in the published papers. 13

In the present study, we have evaluated the results of OCB detection and KI in CSF and serum samples obtained from suspected MS cases submitted to investigation with OCB detection.

## METHODS

### Patients

We included patients who underwent a lumbar puncture (LP) for CSF analysis as part of the MS diagnosis workup. All the LP and CSF analyses were performed at Senne Liquor Diagnóstico, a laboratory specialized in CSF collection and analysis. All of the patients were submitted to a CSF examination as part of the MS diagnostic workup, but none of them underwent lumbar puncture only because of the present study. The procedures for CSF collection and analysis, including the investigation of the intrathecal synthesis of IgG, were those routinely adopted at Senne Liquor Diagnóstico. All tests and assays used in the present study are registered with Brazilian health regulatory agencies. The present study was approved by the institutional ethical board and written free and informed consent was obtained from each patient.

### Intrathecal immunoproduction assessment

The detection of OCBs was performed by isoelectric focusing (IEF), in which unconcentrated CSF and serum were placed on a polyacrylamide gel (5%) and immunoelectrophoresis was performed for 2 hours (1.08 kV; 15 mA; 30–40 W).

The CSF and serum KFLCs were determined using the Freelite Assay (The Binding Site Group, Birmingham, United Kingdom) and the turbidimetric Optilite analyzer (The Binding Site Group). The assay detection limits were of 0.27 mg/L for KFLCs. The KFLC results were expressed through the KI using the following formula: (CSF KFLC/serum KFLC)/(CSF albumin/serum albumin). The KI was considered positive when it was above 5.8.

### Data analysis

Patients in whom the presence of OCBs of types 2 or 3 patterns were classified as OCB+ while the others, as OCB-. Patients with KI > 5.8 were considered KI + . The OCB and KI results were registered, the percentages of concordant (both positive and both negative) and discordant (positive only through one of these two methods) results were recorded, and the significance of these associations was assessed using the Chi-squared test.

## RESULTS

In total, 171 serum and CSF samples from 171 patients were included in the analysis. The mean age of the patients was of 40 ± 14.2 years; 18.9% of them were male, and 81.1% were female. The mean CSF white blood cell count was of 2.5 ± 3.6 cells/mm ^3^ , and the mean protein, glucose, and lactate concentrations were of 29.8 ± 10.2 mg/dL, 58.2 ± 12.4 mg/dL, and 13.8 ± 2.9 mg/dL respectively.

**Figure 2:**
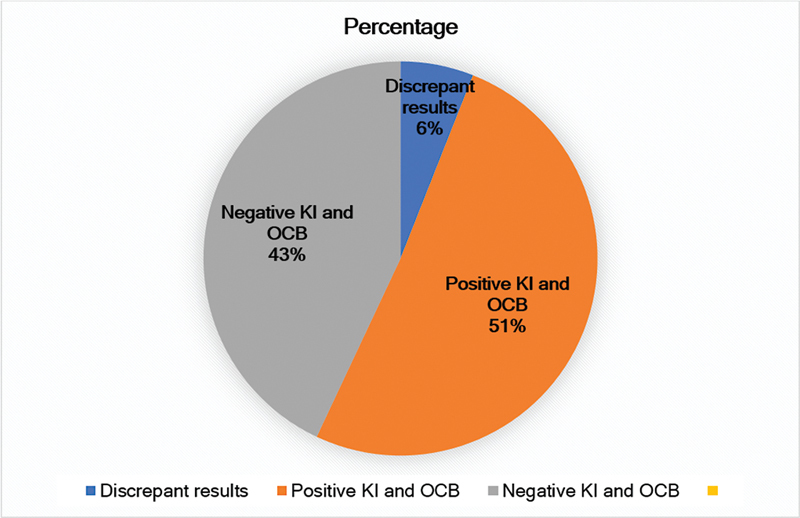
Concordant and discrepant OCB detection and Kappa index (KI) results.

The OCB and KI results were concordant in 161 (94.2%) patients; in 74 (43.3%), both were positive, and in 87 (50.9%), both were negative ( Figure 2 ). In 10 cases: the results were discrepant: KI+ and BOC- in 8, and BOC+ and KI- in 2 ( Table 1 ). The frequency of KI+ results among OCB+ patients and the frequency of KI- results among OCB- patients was statistically significant ( *p* < 0.00001).

**Table 1:** Positive and negative results of the detection of oligoclonal bands (OCBs) and Kappa index (KI) determination (through the Chi-squared test; *p* < 0.00001)

	KI positive	KI negative	Total
**OCB positive (n)**	74	**2**	76
**OCB negative (n)**	**8**	87	95
**Total (n)**	82	89	171

## DISCUSSION

The diagnosis of MS is challenging, as there is no single and definitive marker. The current diagnostic criteria require dissemination in time (different central nervous system [CNS] lesions at different times) and dissemination in space (different CSN lesions at distinct CNS topographies), as demonstrated by a clinical evaluation or by magnetic resonance imaging (MRI) scans. 4 The presence of OCBs, indicating the intrathecal synthesis of IgG, contributes to the diagnosis by replacing the need of a new clinical or radiological lesion for the definition of dissemination in time. 4 However, OCBs are not found in ∼ 5% of MS cases. 4 Indeed, IEF is a laborious and time-consuming technique which can be difficult interpret visually. Therefore, it is reasonable to assess other methods of detecting intrathecal IgG synthesis.

In the present study, the KI results presented a high and statistically significant concordance rate with OCB results, showing that the probability of obtaining the same result with the detection of OCBs and KI is extremely high. This finding is in line with those of previous studies 13 14 15 16 17 18 that have shown that the KI has diagnostic efficacy similar to that of the OCBs. In a previous study by our group 19 on the sensitivity of quantitative methods in relation to OCBs, we identified that other qualitative evaluation methods for IgG synthesis presented a low concordance rate with OCBs. The results of the present study suggest that the KI has higher concordance rate with OCB detection than other quantitative methods. The formula (CSF KFLC/serum KFLC)/(CSF albumin/serum albumin) and the cut-off point of 5.8 according to the manufacturer's instructions seem to be suitable, considering the high and significant concordance level with OCBs.

In the present study, it was not possible to accurately assess the sensitivity and specificity of each method, since the detailed clinical and neuroradiological data needed to define the diagnosis according to the current diagnostic criteria were not available, because the study data were exclusively obtained from the CSF laboratory database. Another issue that could have been assessed was the influence of ethnic factors on the sensitivity of KI; however, we did not have access to the patient data. Previous studies 20 have demonstrated differences in the sensitivity of OCB detection in patients with MS according to ethnicity. To our knowledge, the influence of ethnicity on the sensitivity of CSF light chain measurement has not yet been evaluated in patients with MS. 13

Bearing in mind all of these limitations, the question that arises is that of how to interpret all the discrepant results verified in 10 cases. Among them, 8 were KI+ and OCB-. According to the current diagnostic criteria, patients with typical MS symptoms with MRI scans showing only dissemination in space, and OCB-/KI + , would not be diagnosed with MS, but rather with a clinically-isolated syndrome (CIS). However, we propose that, in such a scenario, the KI+ result should perhaps be taken into account, especially if the index is much higher than the cut-off range and if there is no other alternative diagnosis for the condition of the patient. 8 16 Considering this hypothetical scenario, it is reasonable to suggest that, in highly-suggestive cases in which OCB detection is negative or doubtful, the KI can provide clinical contributions, supporting therapeutic decisions. In fact, some authors 11 12 have proposed the inclusion of FLC evaluation in the MS diagnostic criteria.

On the other hand, the two discrepant cases with detection of OCBs and negative KI would not raise additional doubts considering the current diagnostic criteria, which only consider the detection of OCBs. In the same scenario of a patient fulfilling the clinical and neuroradiological criteria for a CIS with dissemination in space but without dissemination in time, an OCB+ result would be sufficient, regardless of the KI result. 6 Future studies applying both methods in parallel, in populations with known clinical and radiological data, might definitively establish the sensitivity and specificity of measuring IgG light chains for the diagnosis of MS.

There are other limitations to the present study. We had not access to clinical and radiological data, so definitive conclusions on the diagnostic performance of KI and OCB detection could not be drawn. Different KI cut-off values were not tested; however, the value adopted seemed adequate, given the high rate of concordance with the OCBs. Another potential limitation is that the time between the last attack and the collection of CSF and blood samples, as well as disease activity, in MS cases, was not known. This may have influenced the results of the light chains in the CSF, as these markers may be affected by the inflammatory activity status of the disease. However, the fact that OCB detection was performed and the KI was established in the same samples in all cases minimizes this interference. 21 22

Despite these limitations, the results obtained are of great relevance, as they represent real-life data obtained in a uniform manner in a laboratory with great expertise in CSF analysis.

The use of the KI as a new diagnostic biomarker in MS has other potential advantages. The KI is faster, laborsaving, rater-independent, and more reliable, since it does not depend on the visual analysis of the bands. A greater standardization of the procedure and interlaboratory reliability determination may further contribute to the definitive establishment of this method as an alternative and effective way of determining intrathecal IgG production.

In conclusion, the present study supports previous studies that reported that the KI is a good alternative CSF method for MS diagnosis. Considering that the KI is faster and easier to be performed, it may increase the access to the determination of intrathecal IgG immunoproduction (since not all laboratories can perform BOC detection), and make this determination more reproducible.
